# Three-Dimensional Bioprinting of Strontium-Modified Controlled Assembly of Collagen Polylactic Acid Composite Scaffold for Bone Repair

**DOI:** 10.3390/polym16040498

**Published:** 2024-02-11

**Authors:** Weiwei Sun, Wenyu Xie, Kun Hu, Zongwen Yang, Lu Han, Luhai Li, Yuansheng Qi, Yen Wei

**Affiliations:** 1 Beijing Engineering Research Center of Printed Electronics, Institute of Printing and Packaging Engineering, Beijing Institute of Graphic Communication, Beijing 102600, China; 2 National Engineering Laboratory for Modern Silk, College of Textile and Clothing Engineering, Soochow University, Suzhou 215123, China; 3 Department of Chemistry, Tsinghua University, Beijing 100084, China

**Keywords:** 3D bioprinting, controlled assembly, mineralized collagen, PLA, bone tissue engineering

## Abstract

In recent years, the incidence of bone defects has been increasing year by year. Bone transplantation has become the most needed surgery after a blood transfusion and shows a rising trend. Three-dimensional-printed implants can be arbitrarily shaped according to the defects of tissues and organs to achieve perfect morphological repair, opening a new way for non-traumatic repair and functional reconstruction. In this paper, strontium-doped mineralized collagen was first prepared by an in vitro biomimetic mineralization method and then polylactic acid was homogeneously blended with the mineralized collagen to produce a comprehensive bone repair scaffold by a gas extrusion 3D printing method. Characterization through scanning electron microscopy, X-ray diffraction, and mechanical testing revealed that the strontium-functionalized composite scaffold exhibits an inorganic composition and nanostructure akin to those of human bone tissue. The scaffold possesses uniformly distributed and interconnected pores, with a compressive strength reaching 21.04 MPa. The strontium doping in the mineralized collagen improved the biocompatibility of the scaffold and inhibited the differentiation of osteoclasts to promote bone regeneration. This innovative composite scaffold holds significant promise in the field of bone tissue engineering, providing a forward-thinking solution for prospective bone injury repair.

## 1. Introduction

Since the advent of medicine, the repair of various human tissues and organs has been a hot topic of medical research, and the repair of bone defects is one of them [1]. Bone tissue engineering is an important branch of tissue engineering and has great potential for development [2]. As the incidence of bone defects continues to increase [3,4], materials for bone repair have also become a hot topic in materials science research. The commonly used method for bone defect repair is to fix the fracture or bone defect site in a stable spatial environment and make the defect site complete self-healing through the body’s own repair ability. However, this method has some limitations, as it can show a good repair effect for small bone defect areas, but the repair effect is not good when encountering large bone defect areas. Therefore, for the repair of large bone defects, it is necessary to introduce bone repair materials to speed up the self-repair of the body or directly use bone replacement materials for treatment [5].

The rapid development of 3D printing technology in recent years offers good possibilities to optimize the preparation of traditional bone substitutes, aiding in the control of the pore interconnectivity, pore size, and overall porosity of bone repair materials [6,7,8]. However, three-dimensional scaffold structures made of a single biomaterial have drawbacks, such as poor biological activity, insufficient mechanical performance, and inconspicuous bone induction. When two or more biomaterials are organically combined, composite materials not only maintain the relative independence of the properties of the different components but also complement each other’s strengths and weaknesses. This significantly overcomes the drawbacks for using a single material. An ideal bone repair scaffold should not only provide structural support for implanted cells and possess ideal mechanical performance but also offer excellent osteogenic activity to regulate cell responses and promote bone tissue repair [9].

Wolff’s law [10] states that the growth and absorption of the human skeletal system are determined by the mechanical loading conditions on the bones. The compressive strengths of cortical bone and cancellous bone should fall within the ranges 180–213 MPa and 1.5–45 MPa, respectively [11]. When the stress on implanted scaffolds is too low, the scaffolds cannot adequately support bones. If the stress on the implanted scaffold exceeds this range, the parts with higher stiffness will bear more load than the bones, leading to stress shielding. Bone will be resorbed by osteoclasts when the stress on the bone is below 1–2 MPa. The rate of bone formation in the human body is lower than the rate of bone resorption, resulting in decreased bone strength and further leading to osteoporosis. Both the intensity and duration of the mechanical stress affect the activation capacity of osteoclasts. Therefore, excess or insufficient mechanical stress can disrupt the dynamic balance of human bone cells. To ensure effective protection and support, bone scaffolds need to possess a certain level of strength.

Mineralized collagen (MC) [12] is composed of type I collagen (COL-I) and hydroxyapatite (HAP), which mimics the chemical composition and microstructure of the natural bone matrix, in which HAP nanocrystals grow in a periodic and ordered manner on the surface of collagen fibers [13,14]. In previous studies, researchers found that MC has excellent biocompatibility and osteogenic ability, and therefore is widely used for the repair of various bone defects, as well as to promote in situ bone regeneration [15]. Strontium plays a crucial biological role in the skeletal system and has been used as a therapeutic agent for treating conditions that lead to bone defects. For instance, the oral medication strontium ranelate reduces the risk of vertebral fractures in patients with osteoporosis [16,17]. When taken together with strontium lactate and calcium supplements, it can enhance bone mineralization [18]. Strontium exhibits a dual action for inhibiting bone resorption and promoting bone formation. It stimulates the proliferation and formation of osteoblasts while inhibiting the formation of osteoclasts and their mediated bone resorption. It also stimulates blood vessel formation, contributing to the positive effects of strontium-modified porous scaffolds as biomaterials for treating bone defects. Understanding the critical functions of strontium in the skeletal system, researchers have found that the activation of the calcium-sensing receptor and its mediation of cell functions play a crucial role in the mechanism of strontium. Currently, strontium is being studied for incorporation into various materials to treat osteoporosis and promote bone regeneration. These materials include bioactive silica glass [19,20], calcium silicate ceramics [21], and calcium phosphate ceramics [22,23].

Polylactic acid (PLA) is a biodegradable material with excellent performance and belongs to the category of synthetic thermoplastic aliphatic polyesters. It exhibits ideal biocompatibility and possesses good tensile strength and ductility, making it a suitable material for bone tissue engineering. Owing to its outstanding performance, PLA has significant development potential in the field of bone repair materials. Researchers both domestically and internationally have employed various methods to modify and composite PLA materials. Examples include porous scaffolds, like PLA scaffolds modified with type I collagen [24,25], PLA-poly(ethylene oxide)-poly(propylene oxide)-poly(ethylene oxide) triblock copolymer scaffolds [26], PLA-hydroxyapatite/chitosan-graphene oxide three-dimensional composite scaffolds [27], and PLA/polyethylene glycol three-dimensional porous scaffolds [28], for bone repair. Results indicate that these methods enhance the cell compatibility of the scaffolds. The widespread application of PLA, especially in tissue engineering, highlights its potential as a substitute. However, it still has some deficiencies for tissue-engineering applications. Researchers have made numerous improvements to this material, employing different techniques to continually meet the requirements in the field of bone tissue engineering.

In this study, a composite material comprising collagen fibers and HAP was developed by utilizing bovine COL-I. The COL-I fiber/nanohydroxyapatite composite material was assembled, incorporating 10% strontium ions to replace calcium ions to prepare strontium-doped mineralized collagen (SrMC). This material was then uniformly mixed with PLA in 1,4-dioxane for printing. A composite scaffold model was designed according to the requirements of bone repair scaffolds, and a 3D printer was used to create a porous and interconnected composite scaffold with a specific pore size. The scaffold was subjected to surface morphology observation, compression performance testing, cytotoxicity evaluation, assessment of the alkaline phosphatase (ALP) activity in osteoblasts, and the inhibitory effect on osteoclast growth. The porous structure of the scaffold facilitated cell migration, nutrient transfer, bone ingrowth, and blood vessel formation [15]. The results demonstrated that the fabricated composite scaffold showed promising biological properties and potential applications in bone defect repair and promoting in situ bone regeneration.

## 2. Materials and Methods

### 2.1. Materials

Strontium chloride (SrCl_2_), calcium chloride (CaCl_2_), hydroxyapatite, and 1,4-dioxane were purchased from Beijing InnoChem Science & Technology Co., Ltd. (Beijing, China). End-capped poly(L-lactic acid) (PLLACOOR, with a specification of 2.15 dL/g and a molecular weight of around 30,000) was obtained from Jinan Daigang Biotechnology Co., Ltd. (Jinan, China). Acid-soluble type I collagen gel was provided by the Institute of Health Equipment Research, Academy of Military Medical Sciences, Tianjin, China. All the reagents were of analytical grade. The purchased chemical reagents were used directly without further physical or chemical treatment, and deionized water was used in the experiments. RAW 264.7 mouse monocyte macrophages were provided by the Academy of Military Medical Sciences. The cell-counting kit-8 (CCK-8) experiment was conducted using a kit obtained from Monitor Technology Co., Ltd., Shanghai, China. Receptor activator of NF-κB ligand (RANKL) and mouse macrophage-colony-stimulating factor (M-CSF) were purchased from Abcam Trading (Shanghai) Co., Ltd. (Shanghai, China). The mouse TNFSF11/RANKL ELISA kit (96T) and mouse osteoprotegerin/TNFRSF11B ELISA kit (96T) were obtained from Proteintech Group, Inc. (Rosemont, IL, USA).

### 2.2. Preparation of the MC and SrMC Powder

According to a previous report [28], MC powder was prepared by in vitro biomimetic mineralization. Soluble calcium and phosphate were added to an acidic collagen solution and mixed. Sodium hydroxide solution was slowly added dropwise to increase the pH, which caused the calcium and phosphate ions to nucleate and grow into nano-hydroxyapatite arranged along the collagen fibers. When the pH of the mixture approached neutral, the MC precipitated. The resulting MC was washed with water and lyophilized for 48 h. After grinding, it was passed through 200–300 mesh screens to obtain MC powder. SrMC powder was prepared in the same way using SrCl_2_ instead of 10% CaCl_2_ (The molar ratio of Sr^2+^ to Ca^2+^ was Ca:Sr = 9:1). Similarly, 30% and 50% SrMC powders were prepared.

### 2.3. Preparation of the MC/PLA Powder and SrMC/PLA Powder

Taking 10% MC/PLA as an example, MC powder and PLA powder were added to an empty beaker in a mass ratio of MC:PLA = 1:9. Then, 1,4-dioxane was added, and the powders were dispersed in the solution in a fume hood, with magnetic stirring for 4 h until the system was homogeneous. The dispersion was poured into a polytetrafluoroethylene mold and placed in a freezer at −20 °C for 6 h for rapid freezing and molding. The 1,4-dioxane was fully removed by freeze-drying for 24 h, and the homogeneous and fluffy porous material was cut into 1–2 mm pieces and dried under vacuum in a vacuum-drying oven for 7 days to further remove the 1,4-dioxane and obtain a homogeneous 10% MC/PLA powder for 3D printing. Finally, the powder was bottled and sealed. Compared with dichloromethane and trichloromethane dispersants, 1,4-dioxane resulted in the mineralized collagen powder being less likely to settle and the mixture being less likely to be uneven and difficult to shear after freeze-drying. The different-component powders for 3D printing were prepared in the same way. The obtained 3D printed scaffolds were respectively labeled as PLA, 10% MC/PLA, 10% SrMC/PLA, 30% MC/PLA, 30% SrMC/PLA, 50% MC/PLA, and 50% SrMC/PLA according to the MC or SrMC content in the composite scaffolds.

### 2.4. Preparation of the MC/PLA Scaffold and SrMC/PLA Scaffold

The appropriate 3D modeling software (3dsmax2012) was used to design a cylindrical support 1 cm in diameter and 1.6 cm in height, and a multilayer support was designed with an aperture size of 400 μm and all interoperable pores. The powder was loaded into the heating cylinder, sealed with nitrogen gas as a protective gas to adjust the air pressure to 0.55 MPa, and the bottom plate temperature was adjusted to 45 ± 0.5 °C. We waited for the nozzle temperature to be heated to 190 ± 0.5 °C and for about 5 min to ensure that the internal powder melted. Then, the appropriate printing speed and filament speed were adjusted to ensure smooth and uniform filaments formed without voids. The designed model was introduced and printed using a 3D printer to prepare a good 3D stent. The scaffold printed by this method avoids the adverse effects of secondary high temperatures on the PLA material and avoids the problem of the low MC content caused by the wire extrusion process in the early stage, which enabled the MC content to be up to 50%. At the same time, the composite material is mixed uniformly and avoids the problem of the uneven distribution of the different components of the composite material owing to the extrusion of the wire by the internal screw of the extruder.

### 2.5. Material Characterization

The dried MC and SrMC powders were tested using the Brook D8 ADVANCE X-ray (D/max-2550, Tokyo, Japan)powder diffractometer (XRD), which was performed at 2θ = 10–60° at a scan rate of 5°/min. Thermogravimetric analysis (TGA) was conducted using a TG209C thermal gravimetric analyzer from Netzsch Technology (Chennai, India). Approximately 20 mg of each component’s dried solid powder sample was heated in a nitrogen environment from room temperature to 800 °C at a rate of 10 °C/min, with each experiment repeated three times. The goal was to determine if the proportion of the solid residue in the materials with different MC or SrMC contents aligns with the inorganic component proportion in the composite material. The loss of weight within the printing temperature range was also assessed. Differential scanning calorimetry (DSC) analysis was performed using a DSC200PC analyzer from Netzsch Technology. Samples of around 20 mg of each component’s dried solid powder were tested in a nitrogen environment from 0 °C to 300 °C, with each experiment repeated three times. The surface morphology and cross-sectional features of the materials were observed using scanning electron microscopy (SEM, Hitachi SU-8010, 5 KV, Tokyo, Japan), and elemental analysis was conducted using an X-ray spectrometer. Compression performance testing of the 3D-printed bone scaffolds with different compositions was carried out using a maximum 2500 N electronic universal testing machine from Shanghai Qingji Instrumentation (Shanghai, China). Samples were placed in the center of the metal test platform, and compression experiments were conducted at a speed of 1 cm/min for three repetitions, each with a cylindrical scaffold of the same group. Stress and strain curves were obtained based on the sample dimensions (diameter: 1 cm, height: 1.6 cm). Stress was calculated as force/(π × radius^2^) and strain as displacement/sample height.

### 2.6. In Vitro Cell Toxicity

The CCK-8 assay was used to evaluate the cytotoxicity and cell viability of the composite scaffolds with varying components. Passaged L929 cells were seeded at 100 μL per well in a 96-well plate and pre-cultured for 2 h at 37 °C with 5% CO_2_. After adding 10 μL of CCK-8 solution per well, the cells were further incubated for 2 h. The absorbance at 450 nm was measured, and the relative growth rate (RGR) was calculated as follows:

$$RGR=\left(\frac{Experimental\;group\;average\;absorbance}{Control\;group\;average\;absorbance}\right)\times 100\%,$$

The RGRs for the blank controls and strontium-doped and non-doped composite scaffolds were determined at 1, 2, and 3 days of culture. The experiment was repeated, and the average values were used. Cell toxicity was graded according to the relative growth rate, following the classification methods outlined in GB/T 16886.5-2011 [29].

### 2.7. Biocompatibility Analysis

The different components of the scaffold were sterilized by exposing them to UV light for 60 min. The mouse embryonic osteoblast (MC3T3-E1) cells were co-cultured with the scaffold, and the ALP activity was subsequently measured using an ALP assay kit as a criterion for evaluating the biocompatibility of the scaffold. The cells were cultured in a medium supplemented with 10% fetal bovine serum, penicillin (100 μg/mL), streptomycin (100 μg/mL), and amphotericin (0.25 μg/mL). The cells were cultured at 37 °C in a humidified environment with 5% CO_2_ for 12 h, and they were planted in 96-well plates during logarithmic growth (about 1500 cells per well). The experimental groups were incubated for 1, 3, or 5 days using the scaffolds with same serum and antibiotic as those in the medium, while those with only cells were made as the control group. Finally, the OD value was measured at 405 nm using the ALP method.

### 2.8. Impact on Osteoclast Differentiation

The mouse monocyte macrophage, RAW264.7, selected for this experiment is a commonly used osteoclast precursor cell that can be induced to differentiate into osteoclasts by M-CSF, RANKL (encoded by the TNFSF11 gene), and other factors [30]. Osteoclasts are prominent in bone reconstruction and bone density regulation, and most skeletal diseases are associated with abnormal osteoclast activity. In contrast, the interaction between RANKL and osteoprotegerin (OPG, encoded by the TNFRSF11B gene) can regulate osteoclast proliferation and differentiation and maintain the normal bone reconstruction process [31]. OPG is a competitive inhibitor of RANKL, which inhibits the differentiation of osteoclastic precursor cells toward osteoclasts, reduces their production, and decreases osteoclastic bone resorption. The ratio of the RANKL/OPG expression is an important way to evaluate osteoclast production and activity [32]: the lower the RANKL/OPG ratio, the lower the osteoclast production. The expressions of RANKL as well as OPG in the cell supernatant can be detected using an enzyme-linked immunosorbent (ELISA) assay.

The RAW264.7 cells in good condition were taken out and co-cultured with the scaffold in a cell culture incubator at 37 °C and 5% CO_2_ for 12 h. The medium in each well was aspirated, and differentiation was induced with 1 mL of the medium containing final concentrations of 50 μg/L RANKL and 30 μg/L M-CSF. After induction for 1, 3, and 5 days, the three 24-well plates were removed sequentially, and the medium was discarded and rinsed three times with PBS. Then, 150 μL of the lysis solution was added to each well, which were gently blown several times with a pipette gun, and the cells were lysed on ice for 1 min. After sufficient lysis, the supernatant was centrifuged at 1000 rpm for 5 min and stored. The reagents were configured, and the assay operation was performed according to the ELISA kit instructions. Finally, the OD value was measured at 450 nm.

### 2.9. Statistical Analysis

All the experimental groups were estimated as at least 3 times the mean standard deviation (x + SD, n = 3, 4, 5, …). Statistical analysis was performed using Student’s *t*-test or one-way analysis of variance, and * *p* < 0.05, ** *p* < 0.01, and *** *p* < 0.001 were considered as statistically significant.

## 3. Results and Discussion

### 3.1. Formation Mechanism

Human bone exhibits diverse structures across different scales [33], ranging from macro to micro. Bones consist of 50–70% inorganic components, predominantly HAP, 20–40% organic components (mainly COL-I), 5–10% water, and 3% lipids. The key characteristics of bone include its hardness, flexibility, strength, and resistance to brittleness. Despite having a porous structure, bones maintain a high level of strength. Even with a lightweight composition, bones can effectively support the entire human body. These seemingly contradictory features of bones are achieved through the concerted action of ordered structures at various levels, spanning from molecules to macroscopically visible. Controlled assembly MC [12] is composed of organic COL-I and inorganic HAP, replicating the chemical composition and microstructure of the natural bone matrix. The periodic and ordered growth of HAP nanocrystals on the surface of collagen fibers is achieved by controlling factors such as temperature and pH [13,14]. Previous studies have demonstrated that MC exhibits outstanding biocompatibility and osteogenic capabilities, making it widely applicable in diverse bone defect repairs and fostering in situ bone regeneration [15,34].

**Figure 1:**
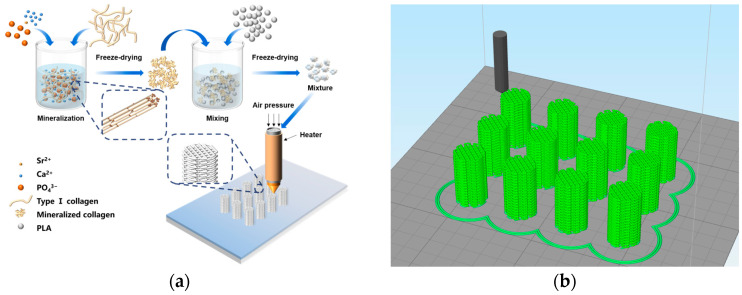
The preparation of a 3D-bioprinted SrMC/PLA composite scaffold: (**a**) material preparation process; (**b**) construction of 3D printed models.

In vitro biomimetic mineralization was employed to prepare strontium-doped controlled-assembly collagen fibers, and the preparation process of the SrMC/PLA composite scaffolds is depicted in Figure 1a. The co-blended powder, after dissolution and freeze-drying in a vacuum-drying chamber to remove 1,4-dioxane, results in SrMC/PLA powder that is suitable for 3D printing, uniform, and easily sheared. Using 3D modeling software, like 3ds Max, a cylindrical scaffold with a diameter of 1 cm and a height of 1.6 cm was created, featuring interconnected multilayered structures with pore sizes of 400 μm, as shown in Figure 1b. The designed model was imported and printed using a 3D printer, producing well-crafted three-dimensional scaffolds. This printing method for scaffolds avoids the adverse effects of secondary high temperatures on PLA materials, prevents the issue of the low MC content due to extrusion-line material problems, and enables the maximum content to reach 50%. Additionally, the composite material is uniformly mixed, avoiding the uneven distribution of the different components in the composite material caused by the extrusion screw inside the extruder.

### 3.2. Characterization Analysis

**Figure 2:**
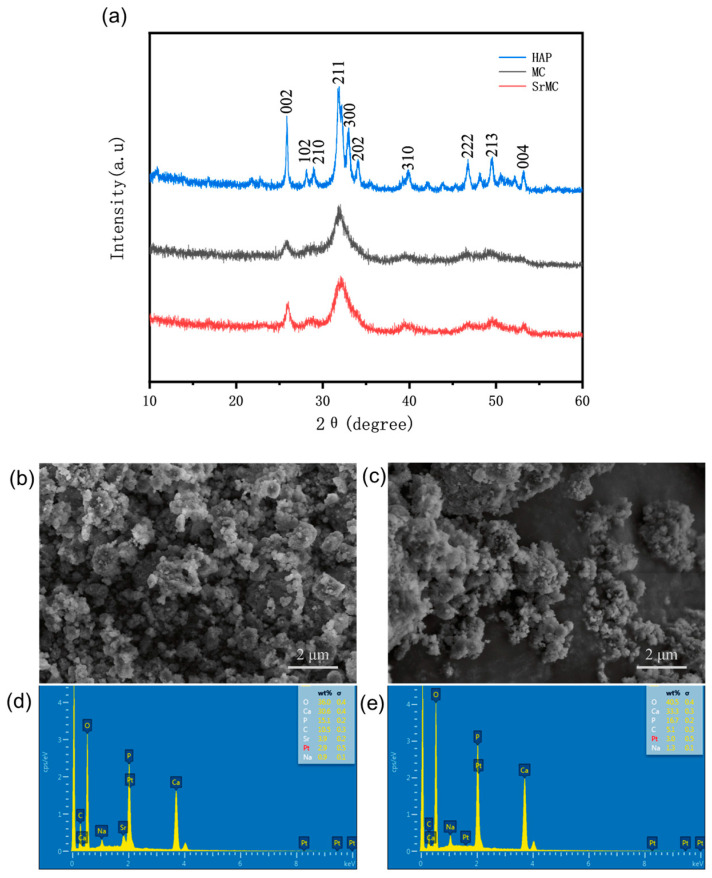
SEM and EDS images of MC powder and strontium-modified controlled-assembly mineralized collagen: (**a**) HAP, MC, and SrMC crystal structural analysis by XRD; (**b**) SEM image of MC; (**c**) SEM image of SrMC.; (**d**) EDS images of MC; (**e**) EDS images of SrMC.

The main inorganic component of human bone is hydroxyapatite. As evident from the XRD results in Figure 2a, the XRD diffraction patterns of the MC and SrMC materials are highly similar to that of hydroxyapatite. The primary component of both the MC and SrMC materials is HAP. The presence of the collagen assembly restricts the crystallization of the HAP crystals in the MC, resulting in smaller HAP crystals in the prepared MC and SrMC materials, which broadens the diffraction peaks for each crystal plane in the XRD pattern. The XRD diffraction spectrum of the SrMC material is similar to that of the MC material, indicating that the preparation of the SrMC in this study did not alter its main structure and retained the characteristic features of the MC.

Through SEM and EDS analysis, we observed the microscopic particle morphologies of MC and SrMC, as shown in Figure 2b–e. It can be observed from the figure that the atomic ratio of Ca:P in the prepared MC is approximately 1.66, while the ratio of (Sr/Ca):P in SrMC is also approximately 1.66. Both ratios closely resemble the proportions of Ca and P in human bones. Additionally, in SrMC, the Sr^2+^:Ca^2+^ ratio is approximately 1:9, in accordance with the expected values, indicating the successful incorporation of strontium ions into this bone repair material.

**Figure 3:**
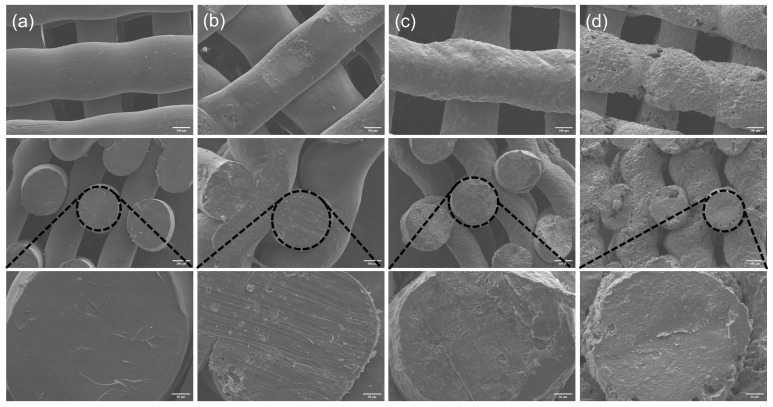
Surface morphologies and cross-sections of scaffolds with different ratios of MC/PLA: (**a**) PLA; (**b**) 10% MC/PLA; (**c**) 30% MC/PLA; (**d**) 50% MC/PLA.

**Figure 4:**
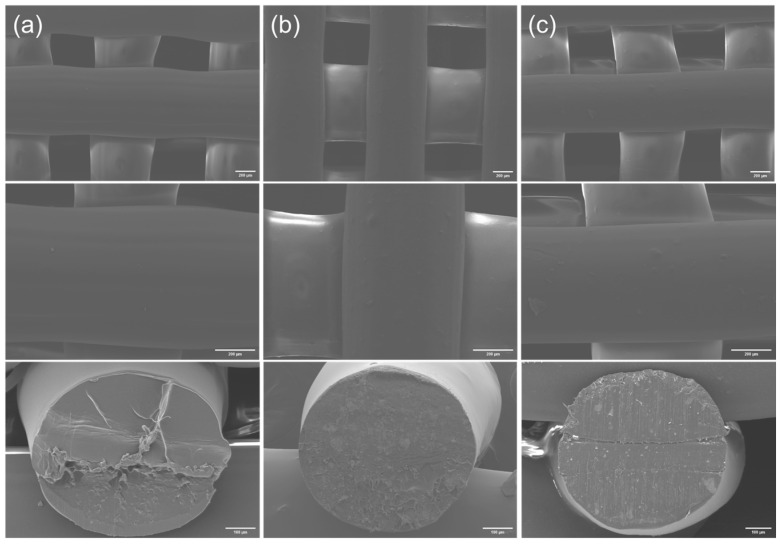
PLA, 30% MC/PLA, and 30% SrMC/PLA scaffold cross-sections and surface morphologies: (**a**) PLA; (**b**) 30% MC/PLA; (**c**) 30% SrMC/PLA.

By observing the surface morphologies and cross-sections of the MC/PLA scaffolds at different ratios (Figure 3), it is evident that all the compositions can be successfully printed. However, as the MC content increases, the printing quality gradually deteriorates. The scaffold with the 50% MC content exhibits cavities and cracks on the surface, and the extruded filaments are prone to collapse. Considering both the printing performance and mechanical properties, a 3D-printed scaffold of SrMC/PLA with a 30% content is recommended. By comparing the surface morphologies and cross-sections of the pure PLA, 30% MC/PLA, and 30% SrMC/PLA scaffolds (Figure 4), it is evident that the SrMC/PLA and MC/PLA scaffolds are not significantly different. Both scaffolds demonstrated favorable 3D printing outcomes, exhibiting regular and undistorted structures. The printed structures appeared orderly. Simultaneously, the pore sizes of the scaffolds range from 400 μm to 500 μm, and the final diameter of each 3D printed strand is around 700 ± 50 μm. All the pores are interconnected, facilitating substance exchange and cell adhesion and growth during the bone repair process. Additionally, this structure is conducive to the subsequent neovascularization and nerve growth [35].

**Figure 5:**
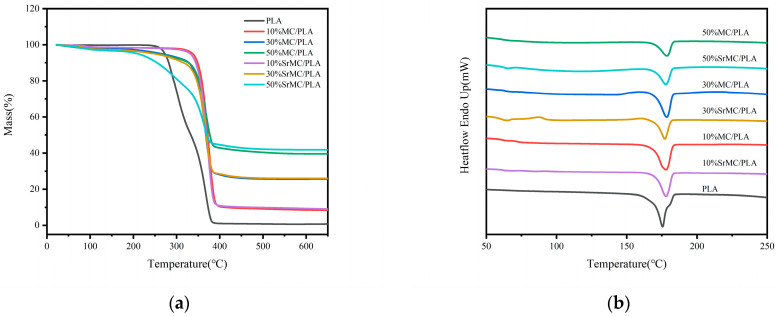
Thermal analysis of composite materials with different compositions: (**a**) TGA curve; (**b**) DSC curve.

TGA, as depicted in Figure 5a, was employed to analyze the contents of inorganic and organic components in the materials. The proportion of the solid residue in the materials with different MC or SrMC contents aligns closely with the proportion of inorganic components in the composite materials. At a temperature of 400 °C, the pure PLA shows no residue, while the other components exhibit residues consistent with expectations. Furthermore, below the printing temperature of 190 °C, the mass loss rate for all the components is less than 5%, indicating the suitability of this material for the specified printing temperature. The printing temperature exerts minimal impact on the material quality, affirming the material’s excellent printability.

The DSC curves, as illustrated in Figure 5b, reveal a melting point range of 175–183 °C. During melting, all the crystals liquefy, and polymer chains assume an irregular state, displaying non-Newtonian flow. The spectra suggest that with an increase in the MC or SrMC content, the melting point of the composite materials slightly elevate. The DSC data indicate that throughout the 3D printing process of this composite material, printing can be conducted at temperatures exceeding 183° C. However, given the molten extrusion stacking principle of the FDM-type 3D printer employed in this study, setting the printing temperature at the melting point of 183 °C may not guarantee complete material melting. Hence, in practical printing, a temperature of approximately 190 °C is set to ensure uniform material extrusion, preventing issues such as bubbles and poor layer-to-layer adhesion and ensuring a successful preparation process.

### 3.3. Mechanical Properties

**Figure 6:**
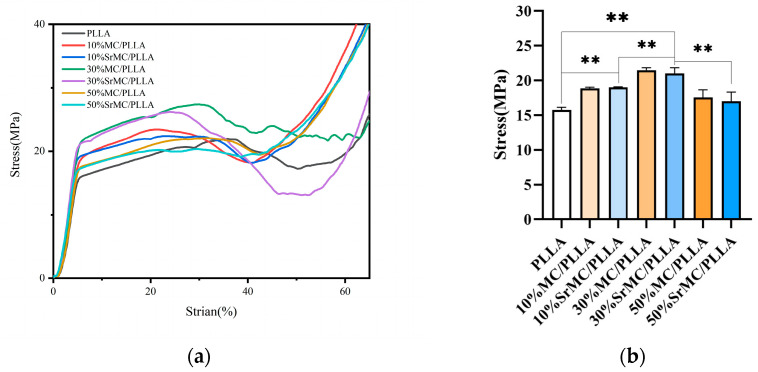
Different scaffold compositions: (**a**) Stress–strain curves; (**b**) Compressive strength bar graph (**: *p* < 0.01).

**Figure 7:**
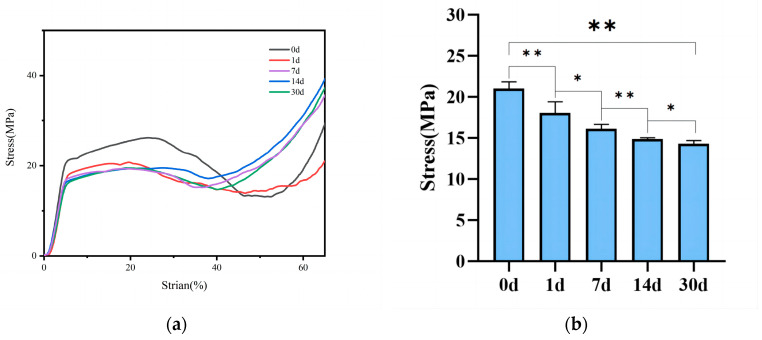
The 30% SrMC/PLA scaffold’s mechanical performance during simulated degradation in biomimetic fluid on different days: (**a**) Stress–strain curves; (**b**) Compressive strength bar graph (*: *p* < 0.05; **: *p* < 0.01).

During compression testing, the scaffold initially undergoes elastic deformation. Once the stress yield value is reached, elastic deformation transitions to non-elastic deformation, with the scaffold’s inherent pores gradually disappearing, and the scaffold ultimately entering a dense phase with the compressive strength continuously increasing. The compressive strength of the scaffold should be equivalent to the stress yield value. From Figure 6, it can be observed that the mechanical performance of the 30% MC/PLA scaffold is optimal, reaching 21.04 MPa, while those of the pure polylactic acid (PLA) are around 15.76 MPa; 10% MC/PLA, approximately 19 MPa; and the 50% group, about 17.01 MPa. These results, after the differential analysis, are statistically significant. At the same contents, the mechanical performance of the SrMC/PLA group is similar to that of the MC/PLA group, with no apparent differences. After immersing the scaffold of the 30% SrMC/PLA group in simulated body fluid for 30 days, as shown in Figure 7, it was observed that the scaffold’s mechanical performance decreases over time. During the 30-day degradation process, the scaffold maintains a compressive strength within the range of that of cancellous bone, without exhibiting stress shielding. In summary, the mechanical performance of 30% SrMC/PLA is optimal, and the mechanical properties remain suitable throughout the 30-day degradation process.

### 3.4. In Vitro Cell Toxicity

Following the assessment of the cytotoxicity using the CCK-8 assay with materials and L929 cells, the experimental results are presented in Table 1. It can be observed that the cytotoxicity grading generally shifted from Grade 1 to Grade 0 after 1 day, 3 days, and 5 days of experimentation. The highest observed cytotoxicity was Grade 1, indicating that the material exhibited no cytotoxicity. After 5 days, all the groups showed a certain relative growth rate compared to the blank group.

### 3.5. Cell Biocompatibility

**Figure 8:**
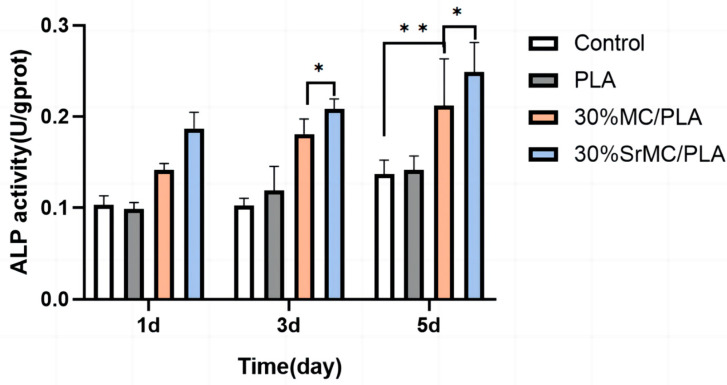
The impacts of different scaffolds on ALP activity in MC3T3-E1 cells (*: *p* < 0.05; **: *p* < 0.01).

According to the comprehensive characterization results and cytotoxicity ratings, the scaffolds with the optimal performance in different groups, specifically those with 30% MC and 30% SrMC additives, were selected for experiments assessing their impact on the ALP activity in MC3T3-E1 cells. The results (Figure 8) indicate that with increasing cultivation time, the ALP activity continuously rises. Notably, the scaffolds with the added MC and SrMC exhibit significantly higher ALP activities compared with those of the pure PLA group and the blank control group (statistically significant, *p* < 0.01). The ALP activity of the 30% SrMC/PLA scaffold is also higher than that of the 30% MC/PLA scaffold (statistically significant, *p* < 0.05), suggesting that the addition of Sr contributes to the increased ALP activity in MC3T3-E1 cells. As ALP is a crucial indicator of early osteogenic differentiation [36], this indicates that the prepared 30% SrMC/PLA scaffold in this study promotes MC3T3-E1 cell proliferation and possesses potential for enhancing osteogenic differentiation.

By combining the above characterization results, the scaffolds with 30% MC and 30% SrMC additions, which had the best performance, were selected from the different groups of scaffolds for the experiments on the effect of the ALP activity on MC3T3-E1 cells. The results in Figure 8 show that the ALP activity increased continuously with increasing culturing time and that the ALP activities of the scaffolds with MC and SrMC additions were significantly higher than those of the pure PLA group and the blank control group (the difference was statistically significant, *p* < 0.01). The ALP activity of the scaffolds in the 30% SrMC/PLA group was higher than that of the scaffolds in the 30% MC/PLA group (the difference was statistically significant, *p* < 0.05), indicating that the addition of Sr favored an increase in the ALP activity in MC3T3-E1 cells and because ALP is an important indicator of early osteogenic differentiation, it indicates that the 30% SrMC/PLA scaffold prepared herein is beneficial to the proliferation of MC3T3-E1 cells and has the potential to promote osteogenic differentiation.

### 3.6. Impact on Osteoclast Differentiation

**Figure 9:**
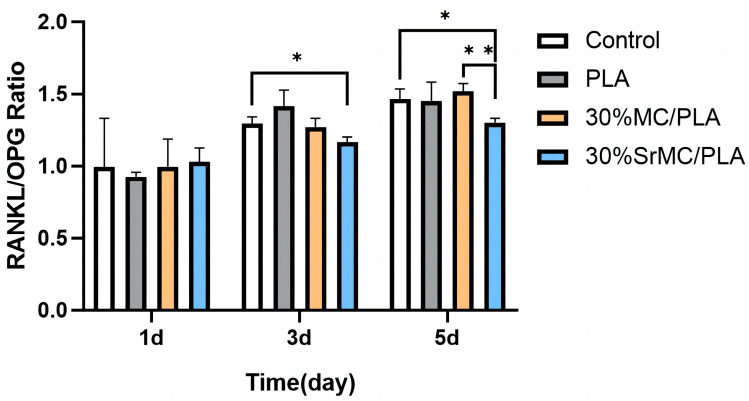
Ratio of RANKL/OPG expression levels for different scaffolds at different days of induced differentiation in RAW264.7 cells (*: *p* < 0.05; **: *p* < 0.01).

As can be seen from Figure 9, the scaffolds in the 30% SrMC/PLA group compared to the blank control group showed a decrease in the RANKL/OPG ratio after 3 and 5 days of induced differentiation by the inducer (the difference was statistically significant, *p* < 0.05), indicating that these scaffolds had a certain inhibitory effect on osteoclast differentiation. Meanwhile, the difference between the 30% SrMC/PLA scaffold and the 30% MC/PLA scaffold was more obvious after 5 days of the induction of differentiation (statistically significant difference, *p* < 0.01), while there was no significant difference between the pure PLA scaffold and the blank control group, indicating that strontium had an inhibitory effect on osteoclastogenesis after being doped into the composite scaffold. The results indicate that the 30% SrMC/PLA composite scaffold prepared in this study is expected to reduce osteoclastic bone resorption to a certain extent, thus achieving better bone repair.

## 4. Conclusions

In summary, in this study, a SrMC/PLA scaffold was successfully prepared for application in bone-tissue engineering. Characterization through XRD analysis and EDS elemental analysis and the observation of the surface morphology of the MC powder revealed that the material possesses an inorganic phase and nanoscale structure similar to those of human bone, making it promising for the fabrication of bone-tissue-engineering repair scaffolds. Thermodynamic analysis indicates that the composite material is suitable for 3D printing and that the temperature near the melting point has no significant impact on the material’s weight loss. Therefore, this composite material is suitable for the 3D printing method used in this study. SEM images reveal that the scaffold’s pore size falls within the range from 400 μm to 500 μm, which is consistent with the model design. All the pores in the scaffold are interconnected, creating a continuous and stable three-dimensional network structure that facilitates material exchange, waste removal, and cell adhesion and growth. The compressive strength of the composite scaffold falls within the stress range of trabecular bone, providing excellent support during the bone repair process without inducing stress shielding. The composite scaffold exhibits good biocompatibility, and the strontium-doped composite material has a certain inhibitory effect on the generation and activity of osteoclasts. Taken together, these characteristics highlight the promising application value of the composite scaffold in bone-tissue-engineering repair materials.

## Figures and Tables

**Table 1:** The OD values, relative growth rates (RGR values), and toxicity ratings for each group after 1, 3, and 5 days of cell cultivation.

Cell Culture Duration	Group	OD Value	RGR Value	Cytotoxicity Classification
1	blank control	0.6003	100.0%	0
	10% MC/PLA	0.7113	118.5%	0
	30% MC/PLA	0.6452	107.5%	0
	50% MC/PLA	0.6240	104.0%	0
	10% SrMC/PLA	0.6382	106.3%	0
	30% SrMC/PLA	0.5697	94.9%	1
	50% SrMC/PLA	0.5415	90.2%	1
3	blank control	0.8661	100.0%	0
	10% MC/PLA	0.8913	102.9%	0
	30% MC/PLA	0.7468	86.2%	1
	50% MC/PLA	0.6928	80.0%	1
	10% SrMC/PLA	0.7968	92.0%	1
	30% SrMC/PLA	0.7461	86.1%	1
	50% SrMC/PLA	0.7969	92.0%	1
5	blank control	1.4585	100.0%	0
	10% MC/PLA	2.1571	147.9%	0
	30% MC/PLA	1.7979	123.3%	0
	50% MC/PLA	1.6432	112.7%	0
	10% SrMC/PLA	1.4741	101.1%	0
	30% SrMC/PLA	1.7311	118.7%	0
	50% SrMC/PLA	1.4585	100.0%	0

## Data Availability

The data are contained within the article.
